# Robotic-assisted laparoscopic excision of a rare primary seminal vesicle leiomyoma

**DOI:** 10.1016/j.eucr.2024.102822

**Published:** 2024-08-12

**Authors:** Kyle Carey, Karina Pique, Sammy A. Shihadeh, Anwar A. Khan, Cian Cranfield, Farzad Esfahani, Brett Parra

**Affiliations:** a Florida State University College of Medicine, 1115 W Call St, Tallahassee, FL, 32304, USA; b Arkansas College of Osteopathic Medicine - Shreveport Campus, 7000 Chad Colley Blvd, Fort Smith, AR, 72916, USA; c HCA Florida West Hospital, 8383 N Davis Hwy, Pensacola, FL, 32514, USA

## Abstract

A primary seminal vesicle leiomyoma (PSVL) is a rare male genitourinary tract tumor. No previous reports have utilized a robotic-assisted laparoscopic posterior approach (RALPA) for surgical management. A 76-year-old man was incidentally found to have a 5cm lobulated mass posterior to the bladder in an abdominal/pelvic computed tomography scan. Biopsy confirmed leiomyoma. RALPA was utilized to excise the mass without complications. PSVLs are benign tumors with obstructive and minimal metastatic potential. Robotic-assisted surgery provides high fidelity, low risk, and an accelerated postoperative course compared to open surgery. The RALPA should be considered when surgical excision of PSVLs is required.

## 1 Introduction

Seminal vesicle tumors are uncommon, usually cystic, and thought to arise from the Müllerian duct. Solid tumors of the seminal vesicle usually represent adenocarcinoma or metastatic invasion from other structures such as the prostate.^1^ This is an important distinction as metastases from malignancies that originate from nearby structures such as the bladder or prostate are considered secondary tumors, which are more common and reveal a different pathological mechanism from primary tumors.^2^ These tumors can be visualized with computed tomography (CT) scans or magnetic resonance imaging (MRI) as a retrovesicular mass within the seminal vesicle, then diagnostically confirmed with a transrectal ultrasound and biopsy. Leiomyomas are benign mesenchymal tumors commonly found in the female reproductive tract. They very rarely occur in males but usually involve the male genitourinary tract or gastrointestinal system.^3^ Few cases of primary seminal vesicle leiomyomas (PSVL) have been published. Lallemand et al. utilized traditional laparoscopic surgery to dissect the seminal vesicles, which appears to be the more common approach.^4^ Robotic-assisted laparoscopy has shown to be superior to traditional methods due to its precision, rapid recovery rates, and ability to reduce blood loss.^5^ We present a case of a rare seminal vesicle mass excised via a robotic-assisted laparoscopic approach not otherwise utilized per the literature.

## 2 Case report

**Fig. 1:**
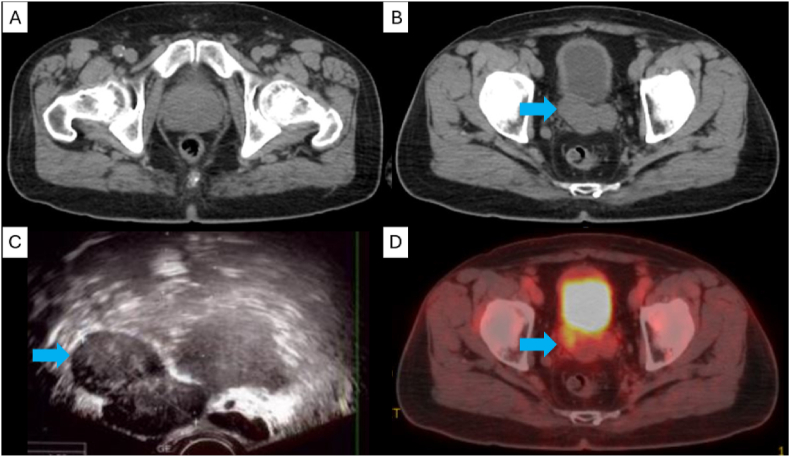
A) Axial CT scan demonstrating benign prostatic hypertrophy measuring 4.2cm × 6.1cm x 5.4cm, without tumor. B) Axial CT scan demonstrating nodular seminal vesicle mass. C) Transrectal ultrasound demonstrating seminal vesicle mass. D) PET-CT demonstrating increased metabolic uptake in the right lateral seminal vesicle.

A 76-year-old man with underlying benign prostatic hyperplasia and no prior cancer diagnosis presented for consultation after incidentally discovering a lobulated mass posterior to the bladder on an abdominal/pelvic CT scan (Fig. 1) during an unrelated emergency department workup. Subsequent abdominal/pelvic MRI and positron emission tomography (PET) (Fig. 1) scan showed a 5cm mass near the seminal vesicles at the base of the prostate gland and posterior to the bladder – representing a primary right seminal vesicle neoplasm.

The patient presented with abdominal discomfort, intermittent urges to defecate, urinary urgency, and urinary frequency. A rectal exam revealed a firm mass adjacent to the prostate. Labs were within normal limits: PSA measured 2.67 ng/ml, testosterone measured 506 ng/dL, and urinalysis was typical. A transrectal ultrasound-guided needle biopsy revealed leiomyoma without prostatic involvement (Fig. 1).

**Fig. 2:**
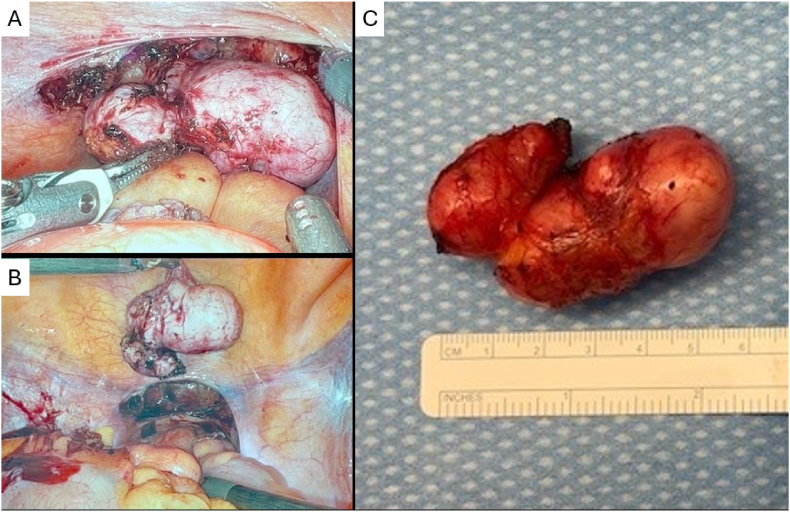
A) Intraoperative photograph: Laparoscopic view of seminal vesicle mass via posterior approach before excision. B) Intraoperative photograph: Laparoscopic view of seminal vesicle mass after excision. C) Gross specimen of seminal vesicle mass, measuring 5.7cm × 2.1cm x 2.1cm, weighing 24 g.

**Fig. 3:**
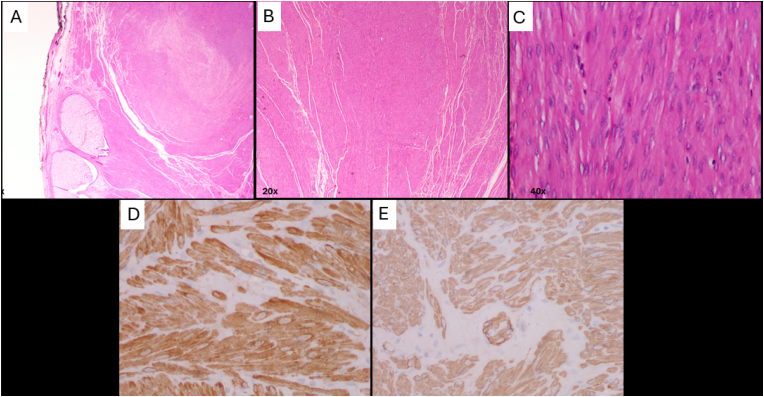
A) Pathology specimen at 2x magnification demonstrating a central portion of well-circumscribed nodular benign smooth muscle-like spindle cell proliferation, with replacement and shifting of seminal vesicles. B) Pathology specimen at 20x magnification, showing microscopic changes consistent with a benign leiomyoma, without signs of hypercellularity or brisk mitotic activity. C) Pathology specimen at 40x magnification, showing microscopic changes consistent with a benign leiomyoma, without signs of hypercellularity or brisk mitotic activity. D) Immunohistochemical stained pathological specimen at 40x magnification demonstrating spindle cell proliferation with positive reactivity for calponin. E) Immunohistochemical stained pathological specimen at 40x magnification demonstrating spindle cell proliferation with positive reactivity for smooth muscle actin.

Due to concern for future enlargement and potential encroachment on the ureters, the patient elected for surgical excision of the seminal vesicle mass. Robotic-assisted laparoscopic surgery was determined to be the optimal and the least invasive option. Using the posterior approach as in that of a prostatectomy, the entire mass was removed sparing the vas deferens, prostate, bladder, ureters, and majority of the seminal vesicles (Fig. 2). Pathologic evaluation of the mass confirmed benign disease (Fig. 3). There were no operative complications. Blood loss was 20 mL. The patient did not endorse complaints or concerning features at the post-operative follow-up visits.

## 3 Discussion

Leiomyomas of the seminal vesicles without prostatic, urinary bladder, or gastrointestinal origin are extremely uncommon. These benign tumors are more commonly present along the uterus, retroperitoneum, gastrointestinal tract, and prostate epididymis.^1^ Their clinical presentation ranges from completely asymptomatic to including pelvic pain/pressure, urinary complaints, and possible bowel obstruction.^6^ The seminal vesicles are coiled, blind-ending tubular structures that give off multiple irregular pouches.^7^ Histologically, they are composed of three layers that include smooth muscle in which the primary leiomyoma is suspected to originate.

Due to the rarity of the disease and low publication volume, the exact prevalence of primary seminal vesicle leiomyomas is unknown. The incidence of primary seminal vesicle tumors described in the literature is less than 100.^8^ However, based on our review of the literature, it can be assumed that these tumors typically arise in patients greater than 50 years (as this appears to be the age with the highest incidence of diagnosis) though they may occur at any age including 10–90 years.^9^

Leiomyomas are rare benign tumors of the male urinary tract with a risk of 0–10 % for transformation into malignancy.^10^ These benign tumors should be differentiated from the more aggressive leiomyosarcomas using MRI and histological evaluation.^11^ Increased uptake on PET scans is commonly seen in benign uterine leiomyomas.^12^ Thus, it is reasonable to assume that while an index of suspicion is appropriate, increased uptake on PET-CT (as in this patient) does not confirm the malignant transformation of PSVLs. Regardless, initial and definitive management of any seminal vesicle mass should include consideration of surgical intervention to confirm the diagnosis and prevent future urinary obstructive complications. Immunohistochemistry staining markers can be used to determine the origin of the seminal vesical tumor, including Wolffian or Mullerian remnants; in particular, CD10 is a marker that differentiates the two while other markers such as PAX8 are less useful due to indiscriminate staining.^6^

Tumors in this region are traditionally excised using a midline laparotomy, with few cases describing a laparoscopic approach, and zero cases to date describing robotic-assisted laparoscopy. In this case, we demonstrate a high fidelity, low risk, and favorable postoperative course when utilizing a robotic-assisted laparoscopic posterior approach compared to the previously documented methods. The posterior approach is the same as that used for robotic-assisted laparoscopic prostatectomy, so most urologists should already be familiar anatomically, proficient, and otherwise comfortable with this approach. Therefore, it can reasonably and easily be applied to practice. Robotic surgery should always be utilized when applicable due to the improved visualization of anatomy, increased dexterity, and improved outcomes for patients. As with prostatectomies, patients should expect a short hospital stay and relatively quick recovery with a resumption of activities within several weeks. Patients should be followed clinically with no additional need for imaging so long as the pathology report is benign and they remain asymptomatic.

## 4 Conclusion

Despite the assumed low incidence of primary seminal vesicle leiomyomas, clinicians should be aware of their occurrence and familiar with updated management strategies. These benign tumors are most likely to be discovered on rectal examination or pelvic imaging and should receive a complete workup to rule out local metastatic disease from adjacent structures. The robotic-assisted laparoscopic posterior approach should be considered (and likely the preferred treatment strategy) compared to the previously reported midline laparotomies or traditional laparoscopic surgery.

## CRediT authorship contribution statement

**Kyle Carey:** Writing – review & editing, Writing – original draft, Supervision, Resources, Project administration, Methodology, Investigation, Funding acquisition, Formal analysis, Data curation, Conceptualization. **Karina Pique:** Writing – review & editing, Writing – original draft, Investigation. **Sammy A. Shihadeh:** Writing – review & editing, Writing – original draft, Investigation. **Anwar A. Khan:** Writing – review & editing, Writing – original draft, Investigation. **Cian Cranfield:** Writing – original draft. **Farzad Esfahani:** Data curation. **Brett Parra:** Writing – review & editing, Supervision, Resources, Methodology, Investigation, Conceptualization.
